# Endothelial glycocalyx injury is involved in heatstroke-associated coagulopathy and protected by N-acetylcysteine

**DOI:** 10.3389/fimmu.2023.1159195

**Published:** 2023-06-06

**Authors:** Na Peng, Yan Geng, Jiafu Ouyang, Shuai Liu, Fangfang Yuan, Yantong Wan, Wenda Chen, Baojun Yu, Youqing Tang, Lei Su, Huaping Liang, Jiang Huai Wang, Jinghua Liu

**Affiliations:** ^1^Guangdong Provincial Key Laboratory of Proteomics, Department of Pathophysiology, School of Basic Medical Sciences, Southern Medical University, Guangzhou, Guangdong, China; ^2^Department of Emergency Medicine, General Hospital of Southern Theater Command, Guangzhou, Guangdong, China; ^3^Department of Gastroenterology, 923 Military Hospital of China, Nanning, Guangxi, China; ^4^Graduate School, Guangzhou University of Chinese Medicine, Guangzhou, Guangdong, China; ^5^Department of Critical Care Medicine, The Third Xiangya Hospital, Central South University, Changsha, China; ^6^Department of Intensive Care Unit, Affiliated Baoan Hospital of Shenzhen, Southern Medical University, Shenzhen, Guangdong, China; ^7^Department of Intensive Care Unit, General Hospital of Southern Theater Command, Guangzhou, Guangdong, China; ^8^State Key Laboratory of Trauma, Burn and Combined Injury, Army Medical University, Chongqing, China; ^9^Department of Academic Surgery, University College Cork, Cork University Hospital, Cork, Ireland

**Keywords:** coagulopathy, endothelial glycocalyx, heat stroke, hyaluronic acid, N-acetylcysteine, syndecan-1

## Abstract

**Introduction:**

Damage to endothelial glycocalyx (EGCX) can lead to coagulation disorders in sepsis. Heat stroke (HS) resembles sepsis in many aspects; however, it is unclear whether EGCX injury is involved in its pathophysiology. The purpose of this study was to examine the relationship between the damage of EGCX and the development of coagulation disorders during HS.

**Methods:**

We retrospectively collected 159 HS patients and analyzed coagulation characteristics and prognosis of HS patients with or without disseminated intravascular coagulation (DIC). We also replicated a rat HS model and measured coagulation indexes, pulmonary capillary EGCX injury in HS rats. Finally, we evaluated the effect of the antioxidant N-acetylcysteine (NAC) on HS-initiated EGCX injury and coagulation disorders.

**Results:**

Clinical data showed that HS patients complicated with DIC had a higher risk of death than HS patients without DIC. In a rat HS model, we found that rats subjected to heat stress developed hypercoagulability and platelet activation at the core body temperature of 43°C, just before the onset of HS. At 24 h of HS, the rats showed a consumptive hypo-coagulation state. The pulmonary capillary EGCX started to shed at 0 h of HS and became more severe at 24 h of HS. Importantly, pretreatment with NAC substantially alleviated EGCX damage and reversed the hypo-coagulation state in HS rats. Mechanically, HS initiated reactive oxidative species (ROS) generation, while ROS could directly cause EGCX damage. Critically, NAC protected against EGCX injury by attenuating ROS production in heat-stressed or hydrogen peroxide (H_2_O_2_)-stimulated endothelial cells.

**Discussion:**

Our results indicate that the poor prognosis of HS patients correlates with severe coagulation disorders, coagulation abnormalities in HS rats are associated with the damage of EGCX, and NAC improves HS-induced coagulopathy, probably through its protection against EGCX injury by preventing ROS generation.

## Introduction

Previous work has shown that patients with HS often display coagulation disorders in the early stage similar to sepsis and the severity of coagulation disorders is closely related to the progression of the disease and prognosis of HS patients (1–4). Emerged evidence has revealed that heat stress, on the one hand, substantially activates the coagulation system and rapidly consumes a large number of coagulation factors, which can directly lead to massive bleeding; on the other hand, the mutual activation of coagulation and inflammatory responses may drive the development of multiple organ dysfunction syndrome (MODS), thus deteriorating the condition of HS patients (5–7). Furthermore, autopsy of patients who died of severe HS has found that extensive micro-thrombosis is the most significant pathological features in these patients (8, 9). Therefore, exaggerated coagulation disorders are considered to be the main cause of death in severe HS patients. However, the precise pathophysiological mechanism(s) involved in the development of severe HS-triggered coagulation disorders is still unclear.

EGCX is an endovascular barrier structure covering the surface of endothelial cells, and its main components include membrane-bound proteoglycans (syndecan and glypican), glycosaminoglycans (CD14, acetaparin sulfate, hyaluronic acid, and chondroitin sulfate), and plasma proteins (albumin and antithrombin), etc., with important physiological functions such as antithrombotic, anti-inflammatory, and regulating vascular permeability. During the development of various diseases such as sepsis and COVID-19, the damage of EGCX leads to the adhesion and aggregation of platelets and neutrophils, and activation of coagulation, which is closely related to the occurrence of coagulopathy. Kobayashi et al. also found that EGCX was damaged in HS rats as manifested by significantly elevated plasma syndecan-1 and was closely associated with the mortality rates of HS rats (10–12); however, whether the occurrence of coagulation disorders in severe HS is associated with the damage of EGCX is not fully elucidated. A number of studies have shown that increased production of reactive oxygen species (ROS) and excessive inflammatory responses are important causes for EGCX injury and coagulation disorders in the development of sepsis (13, 14). Another study suggests that severe HS displays much stronger early inflammatory and oxidative stress responses than sepsis (15). A single-center retrospective study found that compared with severe septic patients, HS patients on admission had much significantly elevated β2-integrin and L-selectin expression on neutrophil, increased ROS release from neutrophils, and enhanced both basal and LPS-stimulated IL-8 release from monocytes and neutrophils (16). Therefore, we speculate that oxidative stress initiated in the early stage of severe HS triggers the inflammatory response and secondary EGCX damage, which ultimately leads to the occurrence of HS-related coagulopathy and disseminated intravascular coagulation (DIC).

In the present study, we first retrospectively analyzed the relationship between the occurrence of disseminated intravascular coagulation (DIC) and prognosis of patients with severe HS. We then examined HS-initiated damage to EGCX and its correlation with coagulation disorders and abnormal inflammatory response in HS rats, and heat-stressed or hydrogen peroxide (H_2_O_2_)-stimulated human umbilical vein endothelial cells (HUVECs). Finally, we evaluated the protective effect of N-acetylcysteine (NAC), an antioxidant and anti-inflammatory agent, on HS-triggered EGCX injury and severe coagulopathy characterized in HS rats.

## Materials and methods

### Clinical data collection

**Figure 1:**
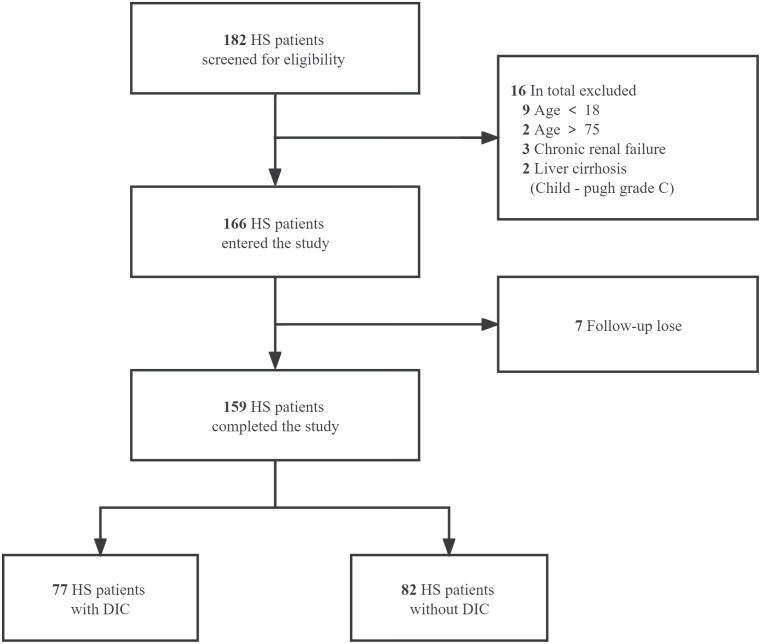
Flow of the enrollment process for HS patients. A total of 182 heat stroke (HS) patients from June 2009 to December 2020 were screened for eligibility. After the exclusion of 16 patients according to the predetermined criteria and 7 patients with data missing on primary outcome, 159 cases were included and divided into HS with DIC and HS without DIC groups.

We retrospectively collected the clinical data from 159 patients (aged 18-75) diagnosed with severe HS who were admitted to the ICU or emergency medicine department of general hospital of PLA southern theatre command from June 2009 to December 2020 and divided into HS with DIC and HS without DIC groups. The diagnosis of HS was based on the Chinese diagnostic criteria for occupational HS (GBZ41-2002) and DIC was diagnosed when the international society on thrombosis and hemostasis (ISTH) score was greater than or equal to 5 (17). Inclusion criteria were age ≥18 years or ≦75 years, and emergency or intensive care unit (ICU) stay for more than 24 h after diagnosis of heatstroke. Exclusion criteria were age < 18 years or >75 years; patients with liver cirrhosis classified as Child-Pugh grade C; patients with chronic renal failure and on renal replacement therapy; other existing irreversible underlying diseases which affect mortality; and patients whose information was missing. The detailed enrollment process of HS patients is summarized in **Figure 1**. This study was approved by the ethics committee of general hospital of PLA southern theater command and informed consent was waived because it was a retrospective observational study.

### Animals model and experimental groups

SPF-grade, 8- to 10-week-old adult male Wistar rats (body weight: 255.3 ± 16.6 g) were purchased from Guangdong medical laboratory animal center (Guangzhou, China). Rats were maintained in the animal center of general hospital of PLA southern theatre command, housed in barrier cages (4 rats per cage) under controlled environmental conditions (12 h light/dark cycle, 55% ± 5% humidity, 23°C), and had free access to standard laboratory chow and water. All experimental protocols involving animals were approved by the institutional animal care and use committee of general hospital of PLA southern theatre command and complied with the animal welfare act.

Rats in the HS group subjected to heat stress were weighed and placed in an artificial high temperature chamber (ambient temperature: 38.5 ± 0.2°C; relative humidity: 60% ± 5%), while rats in the control group were exposed to an ambient temperature of 26.0 ± 0.5°C and a relative humidity of 55% ± 5%. The core body temperature (Tc) and systolic blood pressure (SBP) were measured continuously at an interval of 10 min. Tc was monitored by measuring the rectal temperature using a thermocouple (BW-TH1101, Biowill, Shanghai, China) inserted 6.5 cm from the anus into the rectum. SBP was measured using a noninvasive tail-cuff blood pressure measurement system (Biowill, Shanghai, China). The time point at which SBP started to drop down from the peak level was used as a reference criterion for the occurrence of HS (7, 18, 19). Immediately upon the onset of HS, rats were transferred from the hyperthermic chamber to a 26°C environment with free access to chow and water. In an additional experiment, rats were divided into the control group, HS group, and NAC+HS group. Rats in the NAC+HS group were injected intravenously with 2 ml normal saline containing 150 mg/kg NAC as previous literature through tail vein just before heat stress, and rats in both the control group and HS group received equal volume of normal saline (20).

### Blood and tissue sampling

At 41°C, 42°C, and 43°C of Tc upon heat stress and at different time points (0 h, 2 h, 6 h, 24 h, 72 h, and 216 h) after the onset of HS, rats were anesthetized with 10% chloral hydrate (0.5-1 ml/100 g) and blood samples were collected by cardiac puncture. All animals were sacrificed immediately, and lung tissue samples were harvested and preserved in 2% glutaraldehyde or 4% formaldehyde solution for transmission electron microscopy (TEM) and paraffin section.

### Assessments of standard and viscoelastic coagulation status

Standard (Routine) coagulation indexes including prothrombin time (PT), APTT, and FIB were quantified using a Sysmex CA7000 analyzer (Sysmex, Kobe, Japan). Platelets were counted using a Sysmex XT1800i analyzer (Sysmex). Viscoelastic coagulation parameters including activated clotting time (ACT), CR, and PF were measured using a Sonoclot analyzer (Sienco, Inc., Boulder, CO).

### Visualization of ultrastructure of EGCX by TEM

Harvested rat lung tissue samples for transmission electron microscopy (TEM) were prepared as described previously (21). Briefly, rat lung tissue specimens were fixed in 2% glutaraldehyde and placed a solution containing 2% sucrose, 0.1 M sodium cacodylate phosphate, and 2% lanthanum nitrate at room temperature for 4 h. The specimens were then fixed again in 0.1 M phosphate buffer containing 1% osmium tetroxide at pH 7.4 at 4°C for 1 h, dehydrated in graded acetone, and implanted with propylene oxide into Epon 812 to make ultrathin sections. Ultrathin sections were stained with uranyl acetate and alkaline bismuth subnitrate and visualized under a H-7650 transmission electron microscope (Hitachi, Chiyoda, Japan).

### Detection of syndecan-1 expression by immunofluorescent staining

The paraffin sections of rat lung tissue samples were deparaffinized and hydrated, incubated in 3% H_2_O_2_ at 37°C for 10 min to inactivate endogenous peroxidase, and washed with PBS for 3 times. The sections were then placed in 0.01 M citrate acid buffer solution (pH 6.0) and boiling at high power in a microwave oven for 15 minutes, naturally cooled for more than 20 min to repair antigen, and further incubated with anti-syndecan-1 primary polyclonal antibody (1:100) (Abcam, Cambridge, MA, USA) at 4°C overnight, followed by incubation with goat anti-rabbit Alexa Fluor^®^ 488 immunoglobulin G (IgG) (1:200) (Abcam) for 1 hour at room temperature. The nuclei were stained with Hoche33258. The expression of syndecan-1 in pulmonary vessels was observed by laser confocal microscope (Zeiss, Oberkochen, Germany) and the thickness of syndecan-1 expression in pulmonary vascular endothelial cells was measured by Image J software (NIH, Bethesda, MD, USA).

### Measurement of circulating EGCX biomarkers, coagulation markers, and proinflammatory cytokines

Plasma levels of EGCX biomarkers syndecan-1 and HA were assessed using commercially available enzyme-linked immunosorbent assay (ELISA) kits (Cusabio, Wuhan, China). Plasma levels of coagulation markers von Willebrand factor (vWF), thrombin-antithrombin complex (TAT), plasmin-antiplasmin complex (PAP), and endothelin-1 (ET-1) and proinflammatory cytokines TNF-a and IL-6 were assessed using commercially available ELISA kits (RayBiotech, Guangzhou, China).

### Detection of vWF expression and release in a heat-stressed HUVEC model

HUVECs purchased from Xinyuan Technology (Guangzhou, China) were grown in culture medium containing 10% fetal bovine serum, 4 mM L-glutamine, 100 U/ml penicillin, and 100 μg/ml streptomycin at 37°C in a humidified 5% CO_2_ incubator. The heat-stressed HUVEC model was replicated as described previously (22). Briefly, cells were placed in a circulating water bath at 43°C for 2 h, then replaced with fresh medium, and further incubated at 37°C for 0, 2, 6, 12, and 24 h. In the NAC+HS group, cells were pretreated with 10 mM NAC for 1 h before heat stress. vWF expression in HUVECs was detected using anti-vWF antibody (Abcam) and anti-rabbit Alexa Fluor 488 IgG (Cell Signaling Technology, Veverly, MA, USA) by immunofluorescent staining as described previously (23). vWF levels in the culture supernatant of HUVECs were measured using a vWF ELISA kit (Abcam).

### Assessment of ROS production and release of syndecan-1 and HA in heat-stressed or H_2_O_2_-stimulated HUVECs

Cultured HUVECs were subjected to heat stress as described above or stimulated with 100 μM H_2_O_2_ for 0, 2, 6, and 24 h. In the NAC+HS or NAC+H_2_O_2_ group, cells were pretreated with 10 mM NAC for 1 h before heat stress or exposure to H_2_O_2_. For detection of ROS production, cells in each group were labeled with an ROS indicator, DCFH-DA (Beyotime, Shanghai, China) in the dark for 20 min. After washing with PBS, ROS production in HUVECs, as represented by DCFH-DA positive cells, was detected by FACScan analysis. HUVECs in the control, H_2_O_2_, NAC+control, and NAC+H_2_O_2_ groups were also collected at the indicated time points by centrifugation and fixed in 2% glutaraldehyde for 24 h. Electron microscopic specimens were prepared from the cell pellets to visualize the EGCX under TEM. Syndecan-1 expression was detected by incubating HUVECs in each group with anti-syndecan-1 antibody (Abcam) and anti-rabbit Alexa Fluor 488 IgG (Cell Signaling Technology) by immunofluorescent staining. Syndecan-1 and HA levels in cell supernatants collected from HUVECs at the indicated time points in each group were assessed by their ELISA kits (Abcam).

### Statistical analysis

To analyze the kinetic changes of coagulation function in HS rats, the values of PT, APTT, FIB, PLT, ACT, CR, and PF were converted into percentages of the control group (100% as the baseline) before performing the statistical analysis. All data were tested for normal distribution. Data were expressed as the mean ± standard deviation (SD) if they met the normal distribution or as the median of the interquartile range (IQR) if they did not meet the normal distribution. The Kaplan-Meier curve was used to analyze the impact of the presence of DIC on the prognosis of HS patients. To compare differences between groups, one-way analysis of variance (ANOVA) or nonparametric test followed by multiple comparisons tests were performed. Statistical analyses were conducted using SPSS v.20.0 (IBM Corp, New York, NY, USA) and p<0.05 was considered statistically significant.

## Result

### Coagulation disorders and prognosis of HS patients complicated with DIC

**Figure 2:**
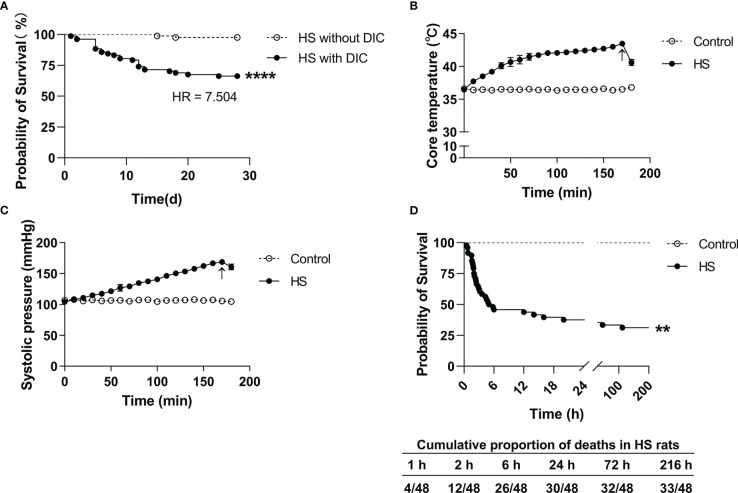
Survival curves in HS patients and HS rats. **(A)** Kaplan–Meier curve shows a significant difference in 28-day survival between heat stroke (HS) patients with DIC (n=77) and without DIC (n=82) (*p*<0.0001). **(B, C)** Changes of core body temperature (Tc) and systolic blood pressure (SBP) in rats subjected to heat stress from 0 min to 200 min with an interval of 10 min in comparison with control rats. Arrows indicate the time point of Tc and SBP starting to fall and the onset of HS. **(D)** Kaplan–Meier curve shows a significant difference in 9-day survival between HS (n=48) and control (n=24) rats (p<0.0001), and the cumulative proportion of deaths in HS rats.

Among the 159 HS patients, 77 (48.4%) had DIC, and 82 (51.6%) had no DIC. As outlined in **Table 1**, HS patients with DIC were older (*p*=0.0258) and had higher maximum Tc (*p*=0.0022) compared with HS patients without DIC. Coagulation parameters including APTT, PT, international normalized ratio (INR) of PT, FIB, fibrin degradation products (FDP), D-Dimer, and platelet count (PLT) in the DIC group were significantly abnormal (all *p*<0.0001 versus the non-DIC group). The length of stay in the ICU and the total length of stay in HS patients with DIC were significantly longer than those in HS patients without DIC (*p*<0.0001). The 28-day mortality rate was 33.77% in the DIC group compared to a 2.44% 28-day mortality rate in the non-DIC group, with a hazard ratio of death at 7.504 between the two groups (*p*<0.001) (**Table 1**; **Figure 2A**). These results indicate that abnormal coagulation is closely related to the poor prognosis of HS patients.

**Table 1:** General information, coagulation parameters, and outcomes in HS patients with and without DIC.

	HS with DIC	HS without DIC	*P -* value
Age (years), median (IQR)	25.00(21.00-38.00) n= 77	22.00 (20.00-26.25) n= 82	0.0258
Sex, (male/female)	(76/1)	(82/0)	0.4843
Maxcore temperature (°C), median (IQR)	40.25 (39.33-41.45) n= 72	39.70(39.00-40.45) n= 77	0.0022
APTT (s), median (IQR)	76.10 (46.50-112.9) n= 77	38.00 (34.48-41.40) n= 82	<0.0001
PT(s), median (IQR)	28.90 (23.35-39.05) n= 77	15.30(14.28-17.03) n= 82	<0.0001
INR, median (IQR)	2.68(1.98-3.87) n= 77	1.22(1.11-1.39) n= 82	<0.0001
FIB (g/L), median (IQR)	1.90(1.20-2.45) n= 77	2.30 (2.00-2.80) n= 82	<0.0001
FDP (μg/ml), median (IQR)	53.50 (21.75-122.70) n= 33	4.00 (4.00-10.48) n= 62	<0.0001
D-Dimer (μg/ml)r, median (IQR)	10.2 (5.7 - 20.0) n= 77	0.63 (0.37-2.70) n= 82	<0.0001
PLT (x10^9^/L), median (IQR)	43.00 (28.50-68.50) n= 77	171.0 (132.8-207.3) n= 82	<0.0001
APACHE II, median (IQR)	15.00(10.00-20.00) n= 77	5.00 (2.75-8.00) n= 82	<0.0001
ISTH Score, median (IQR)	7.00(6.00-7.00) n= 77	2.00 (0.00 - 2.00) n= 82	<0.0001
Length of stay in ICU (days), median (IQR)	11.00 (5.50-17.00) n= 77	4.00 (2.00-5.00) n= 82	<0.0001
Total length of stay (days), median (IQR)	15.00 (9.00-41.00) n= 77	10.00 (4.00-14.00) n= 82	<0.0001
In-hospital death (%)	33.77%(26/51)	2.44%(2/80)	<0.0001

PT, prothrombin time. aPTT, activated partial thromboplastin time. FIB, ﬁbrinogen. PLT, platelet count. INR, international normalized ratio. APACHE II, acute physiology, and chronic health evaluation score II. ISTH, International society on thrombosis and hemostasis. IQR, interquartile range.

### Establishment of a rat HS model with an abnormal coagulation function

**Figure 3:**
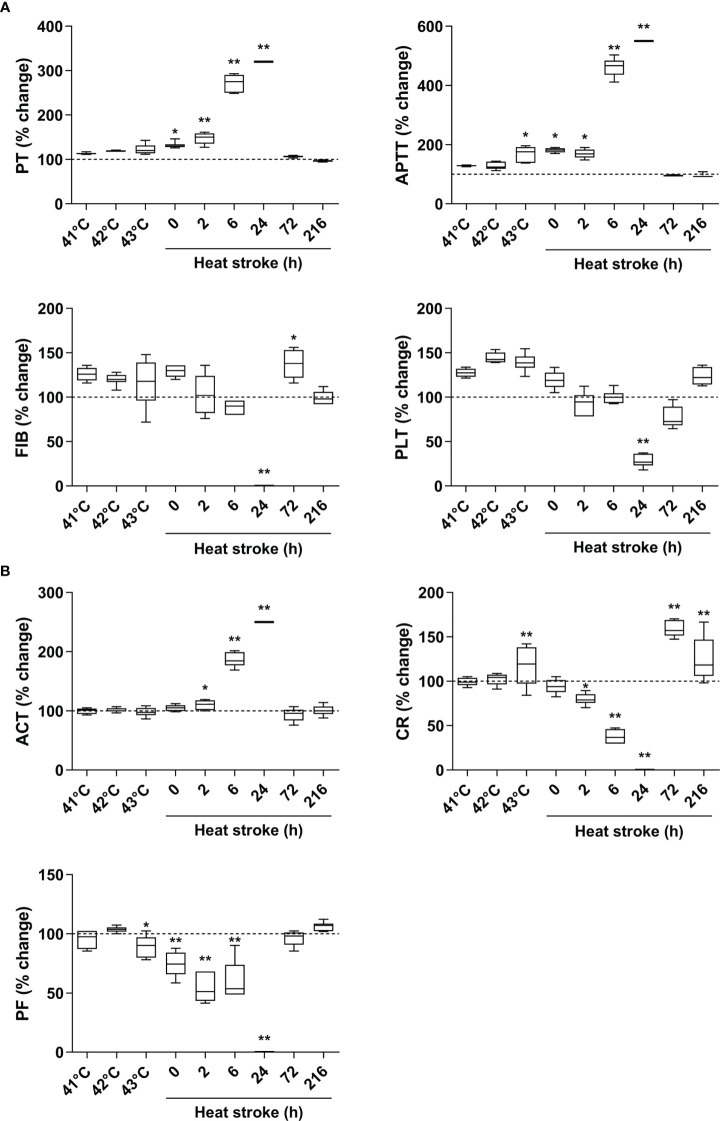
An evolution of abnormal coagulation function is the characteristic in HS rats. **(A)** The standard coagulation parameters PT, APTT, FIB, and PLT were assessed in rats at a Tc of 41 °C, 42 °C, 43 °C and 0, 2, 6, 24, 72, 216 h after HS (n=6 per group). **(B)** The trend of changes in viscoelastic coagulation parameters ACT, CR, and PF was measured in rats at a Tc of 41 °C, 42 °C, 43 °C and 0, 2, 6, 24, 72, 216 h after HS (n=6 per group). Due to the large differences in some time points, values represent as fold changes of the baseline (100%) of the control group to improve aesthetics of graphics. *p<0.05, **p<0.01 compared with the control group. PT, prothrombin time; APTT, activated partial thromboplastin time; FIB, fibrinogen; PLT, platelet count; ACT, activated clotting time; CR, clot rate; PF, platelet function.

Upon heat stress, both the Tc and SBP of rats (n=3) increased gradually from 36.5 ± 0.15°C to 43.5 ± 0.12°C (**Figure 2B**) and from 104.3 ± 2.1 mmHg to 168.7 ± 3.1 mmHg (**Figure 2C**), respectively. At 170 min of heat stress, the SBP of rats began to decrease (**Figure 2C**) and that time point when the SBP begin to decrease was defined as the occurrence of HS as described previously (7, 18, 19). The mortality rate of HS rats was 25.0%, 54.2%, and 62.5% at 2 h, 6 h, and 24 h after the onset of HS (n=48), respectively (**Figure 2D**), which is consistent with the previously reported mortality rates in a dog model of severe HS (24). We further examined the coagulation function in rats subjected to heat stress at different time point (n=6 in each subgroup). Following heat stress, both PT and APTT prolonged gradually, and reached a statistically significant difference either at 0 h of HS or at the Tc of 43°C (*p*<0.05 versus control rats) (**Figure 3A**). Notably, the prolongation of PT and APTT reached their peak levels at 24 h of HS, with a 3.2-fold increase and a 5.5-fold increase, respectively (*p*<0.01 versus control rats), and then entered the recovery phase (**Figure 3A**). Before the occurrence of HS i.e., at the Tc of 41°C, 42°C, and 43°C, both FIB and PLT were higher than those in the control group, but had no statistically significant differences; however, FIB and PLT started to decrease after the commence of HS and reached the lowest levels at 24 h of HS (*p*<0.01 versus control rats) (**Figure 3A**). These results suggest that the coagulopathy of HS is completely decompensated within 24 h after the onset of HS. ACT, which reflects the function of coagulation factors, was significantly prolonged at 2 h of HS (*p*<0.05 versus control rats) and reached its highest level at 24 h of HS (*p*<0.01 versus control rats) (**Figure 3B**), indicating a gradually aggravated hypo-coagulable state in HS rats. CR, which represents the rate of clot formation, elevated significantly at the core body temperature of 43°C (*p*<0.01 versus control rats), then began to decline from 2 h of HS, and finally reached the lowest value at 24 h of HS (*p*<0.01 versus control rats) (**Figure 3B**). PF decreased gradually from the Tc of 43°C and reached its minimum level at 24 h of HS (*p*<0.01 versus control rats) (**Figure 3B**). All these kinetic changes in ACT, CR, and PF reflect the massive consumption of coagulation components during the process of coagulation activation.

### Elevated circulating coagulation markers and proinflammatory cytokines in HS rats

**Figure 4:**
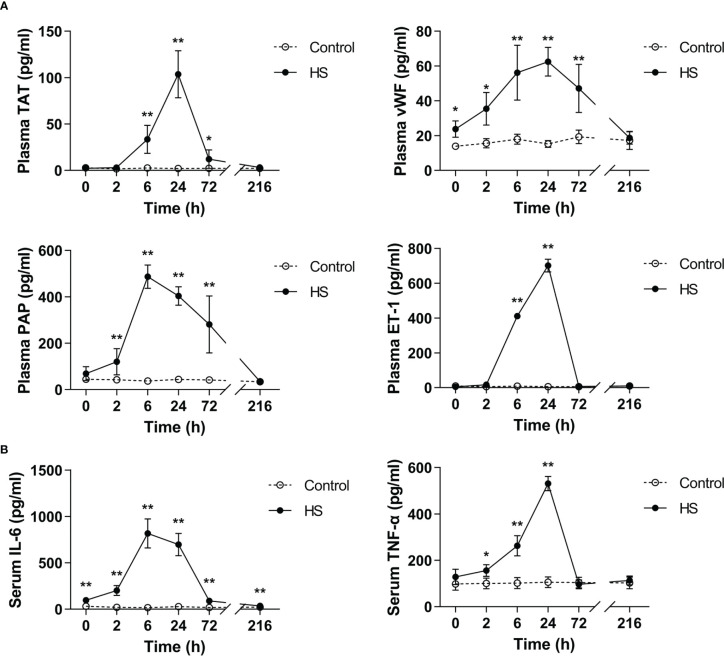
Substantially elevated circulating coagulation-related biomarkers and proinflammatory cytokines in HS rats. **(A)** Plasma levels of coagulation-related biomarkers TAT, vWF, PAP, and ET-1 were assessed at 0, 2, 6, 24, 72, and 216 h post HS. **(B)** At the indicated time points, serum IL-6 and TNF-α concentrations were detected at 0, 2, 6, 24, 72 and 216 h post HS. Data are presented as mean ± SD from six rats per time point. *p<0.05, **p<0.01 compared with the control group. TAT, thrombin-antithrombin complex; vWF, von Willebrand factor; PAP, plasmin-antiplasmin complex; ET-1, endothelin-1.

TAT, vWF, PAP, and ET-1 are well-recognized markers reflecting coagulation activation, platelet activation, fibrinolytic disorders, and endothelial cell damage (25–27). As shown in **Figure 4A**, plasma TAT, vWF, PAP, and ET-1 elevated sharply after the commence of HS (*p*<0.05, *p*<0.01 versus control rats), reached their peak levels at 6 h of HS for instance plasma PAP or at 24 h of HS for instance plasma TAT, vWF, and ET-1 (*p*<0.01 versus control rats), and thereafter began to decline. Serum TNF-α increased significantly at 2 h of HS (*p*<0.05 versus control rats), reached its maximum level at 24 h of HS (*p*<0.01 versus control rats), and declined to the level similar to that seen in control rats, whereas serum IL-6 elevated significantly immediately after the onset of HS, reached its peak level at 6 h of HS, and then decreased gradually but was still significantly higher than that in the control group even at 216 h of HS (all *p*<0.01 versus control rats) (**Figure 4B**).

### Damaged pulmonary capillary EGCX and increased plasma syndecan-1 and HA in HS rats

**Figure 5:**
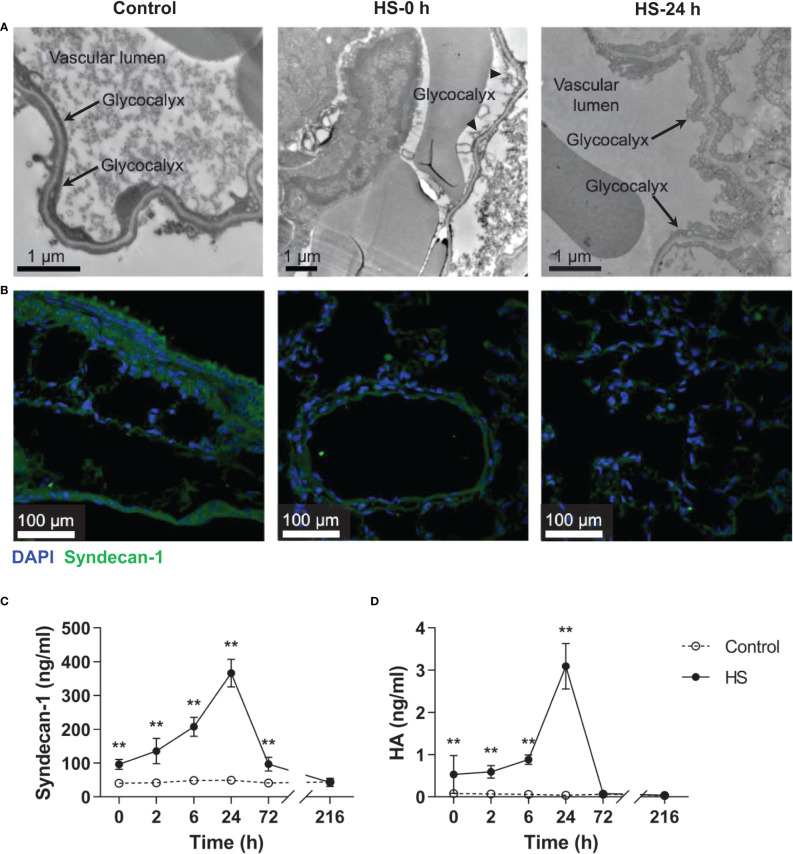
HS rats display severe EGCX damage and increased release of syndecan-1 and HA in the circulation. The lung tissue section collected from control rats and HS rats at 0 h and 24 h post HS was assessed for pulmonary capillary EGCX under transmission electron microscope **(A)** and syndecan-1 expression by immunofluorescent staining with anti-syndecan-1 Ab and Alexa Flour 488-conjugated secondary Ab **(B)**. Cell nuclei were stained with Hoche 33258. Results shown represent one experiment from a total of three separate experiments. Bars represent 1 μm in **(A)** and 100 µm in **(B)**. **(C, D)** Plasma levels of syndecan-1 and HA were measured at the indicated time points post HS. Data are mean ± SD from six rats per time point. **p<0.01 compared with the control group. EGCX, endothelial glycocalyx; HA, hyaluronan acid.

As reveled by TEM, the pulmonary capillary EGCX was intact in control rats, whereas the pulmonary capillary EGCX in HS rats began to shed at 0 h of HS and the shedding of EGCX was more obvious or even absent at 24 h of HS (**Figure 5A**). Immunofluorescent staining showed that syndecan-1 expression, as represented by the green fluorescence, was uniform and continuous in the pulmonary capillary endothelial layer of control rats, indicating an intact EGCX, whereas syndecan-1 in HS rats started to fell off and distributed intermittently at 0 h of HS, and at 24 h of HS syndecan-1 expression was dramatically reduced in a discontinuous state and capillary endothelial layers were structurally disordered (**Figure 5B**). Syndecan-1 and HA are two components of EGCX, and their circulating levels indirectly reflect the integrity of EGCX (28). As shown in **Figures 5C, D**, plasma syndecan-1 and HA levels increased gradually after the onset of HS and reached their peak levels at 24 h of HS (*p*<0.01 versus control rats), indicating an aggravated EGCX damage. By using the Spearman rank test, we found that plasma syndecan-1 and HA levels correlated positively with the circulating levels of coagulation markers including PAP, vWF, TAT, and ET-1, and of proinflammatory cytokines including IL-6 and TNF-α (**Table 2**).

**Table 2:** Correlations of plasma glycocalyx components with coagulation biomarkers and inflammatory cytokines in HS rats.

	Syndecan-1		Hyaluronan (HA)	
	R	*P*-value	R	*P*-value
PAP	0.7668	<0.0001	0.6179	0.0013
vWF	0.9419	<0.0001	0.8235	0.0001
TAT	0.9898	<0.0001	0.8914	<0.0001
ET-1	0.9798	<0.0001	0.8505	<0.0001
IL-6	0.9554	<0.0001	0.6510	0.0006
TNF-α	0.9554	<0.0001	0.8931	<0.0001


PAP, plasmin-antiplasmin complex. vWF, von Willebrand factor. TAT, thrombin-antithronbin complex. ET-1, endothelin-1. IL-6, interleukin-6. TNF-α, tumor necrosis factor. HA, hyaluronan acid.

### Pretreatment with NAC attenuates vascular EGCX injury in HS rats

**Figure 6:**
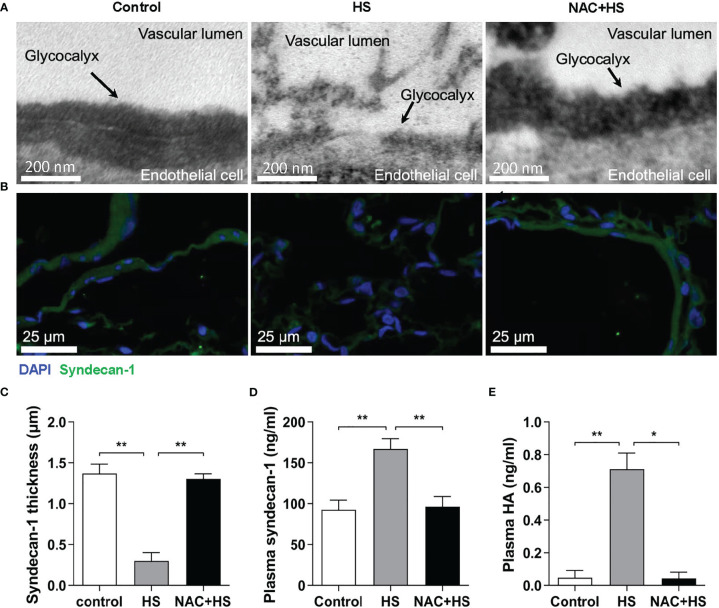
Pretreatment with NAC ameliorates EGCX injury in HS rats. The lung tissue samples collected from the control, HS, and NAC+HS groups were assessed for pulmonary capillary EGCX under transmission electron microscope **(A)** and syndecan-1 expression by immunofluorescent staining with anti-syndecan-1 Ab and Alexa Flour 488-conjugated secondary Ab **(B)**. Cell nuclei were stained with Hoche 33258. Results shown represent one experiment from a total of three separate experiments. Bars represent 200 nm in **(A)** and 25 µm in **(B)**. **(C)** The thickness of syndecan-1 expression was quantitatively analyzed using Image J software. **(D, E)** Plasma levels of syndecan-1 and HA were measured at 2 h post HS between the control, HS, and NAC+HS groups. Data are mean ± SD from six rats per group. *p<0.05, **p<0.01 compared with the HS group. NAC, N-acetylcysteine; EGCX, endothelial glycocalyx; HA, hyaluronan acid.

To ascertain the impact of EGCX injury on HS-associated coagulation disorders, we examined the alterations in vascular EGCX at 2 h of HS upon pretreatment with NAC in HS rats. We particularly selected the time point at 2 h of HS, as HS rats displayed a significantly abnormal coagulation function at this time point. Moreover, the time window within 2 h after the onset of HS is the clinically critical period of treatment for HS patients (29). There was substantial shedding or even absence of EGCX in the pulmonary capillaries at 2 h of HS; however, pretreatment with NAC efficiently alleviated HS-initiated EGCX damage as revealed by TEM (**Figure 6A**). Immunofluorescent staining further showed that syndecan-1 expression in the pulmonary vascular endothelial layer in HS rats was distributed intermittently at 2 h of HS compared with control rats where syndecan-1 expression was continuous and intact; pretreatment with NAC prevented the loss of syndecan-1 (**Figure 6B**) and significantly restored the thickness of syndecan-1 form 0.30 ± 0.10 μm observed in HS rats to 1.23 ± 0.18 μm (*p*<0.01) (**Figure 6C**). Moreover, pretreatment with NAC markedly attenuated HS-induced elevation in plasma syndecan-1 and HA levels from 152.1 ± 36.5 ng/ml and 0.68 ± 0.14 ng/ml to 98.1 ± 9.6 ng/ml (*p*<0.01) and 0.057 ± 0.034 ng/ml (*p*<0.01), respectively, almost same as the levels seen in control rats (**Figures 6D, E**).

### Pretreatment with NAC reduces serum inflammatory cytokines and improves the hypo-coagulable state in HS rats

**Figure 7:**
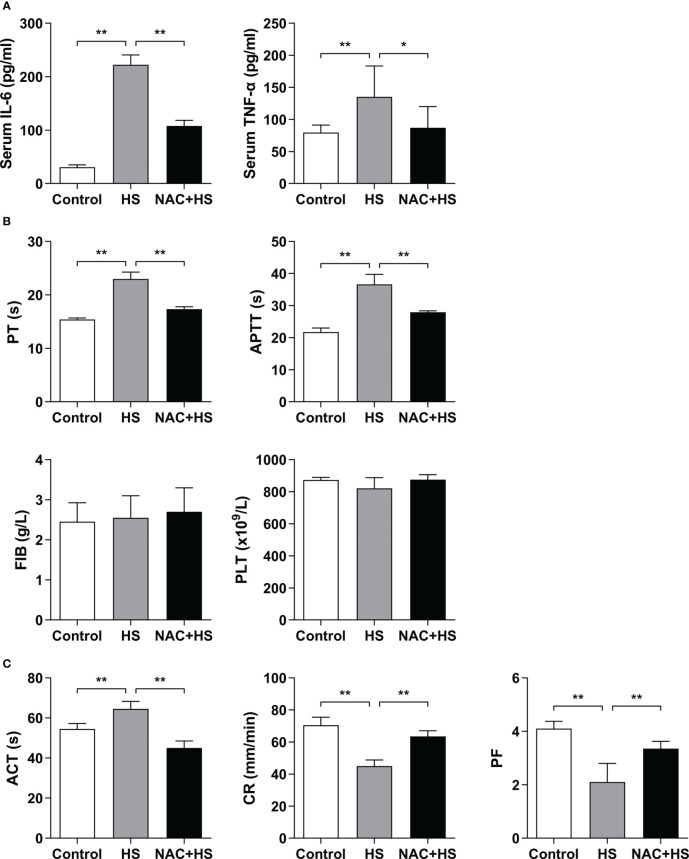
Pretreatment with NAC attenuates proinflammatory cytokine release and improves the abnormal coagulation function in HS rats. Serum IL-6 and TNF-α **(A)**, plasma standard coagulation parameters including PT, APTT, FIB, and PLT **(B)**, and viscoelastic coagulation parameters including ACT, CR, and PF **(C)** were assessed at 2 h post HS. Data are mean ± SD from six rats per group. *p<0.05, **p<0.01 compared with the HS group. PT, prothrombin time; APTT, activated partial thromboplastin time; FIB, fibrinogen; PLT, platelet count; ACT, activated clotting time; CR, clot rate; PF, platelet function.

As shown in **Figure 7A**, serum levels of IL-6 and TNF-α in HS rats were significantly increased at 2 h of HS compared with control rats (*p*<0.01), whereas pretreatment with NAC significantly attenuated HS-induced elevation in serum IL-6 and TNF-α (*p*<0.01 versus HS rats). Furthermore, pretreatment with NAC substantially abated HS-triggered prolongation of both PT and APTT (*p*<0.01 versus HS rats) (**Figure 7B**), and effectively reversed the prolonged ACT and decreased CR observed in HS rats (*p*<0.01) (**Figure 7C**), indicating that pretreatment of HS rats with NAC is capable of preventing coagulation disorders occurred at 2 h of HS. There were no significant differences in PLT and FIB at 2 h of HS among control rats, HS rats, and HS rats pretreated with NAC (**Figure 7B**); however, PF in HS rats was significantly reduced at 2 h of HS (*p*<0.01 versus control rats) and pretreatment with NAC markedly enhanced PF (*p*<0.01 versus HS rats) (**Figure 7C**).

### NAC alleviates EGCX damage and attenuates vWF release from the heat-stressed endothelial cells

**Figure 8:**
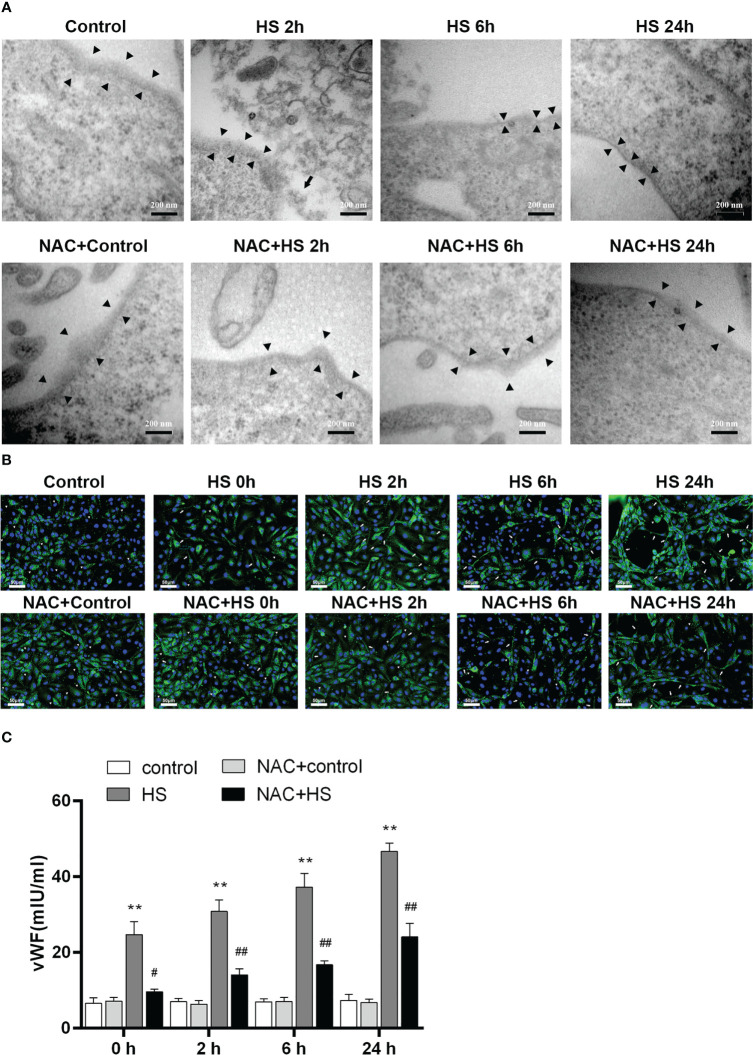
NAC prevents the release of vWF from the heat-stressed vascular endothelial cells by alleviating EGCX damage. **(A)** HUVECs collected from the control, HS, and NAC+HS groups at 2 h, 6 h, and 24 h after HS were assessed for EGCX under transmission electron microscope. Bars represent 200 nm. **(B)** vWF expression was detected by immunofluorescent staining with anti-vWF Ab and Alexa Flour 488-conjugated secondary Ab. Cell nuclei were stained with DAPI. Results shown represent one experiment from a total of three separate experiments. vWF multimers are normally compactly stored in the W-P bodies as graininess (arrowheads), and vWF ribbons or bands (arrows) are formed when VWF is released from the W-P bodies upon HS. Bars represent 50 μm. **(C)** vWF levels in culture supernatants collected from the control, HS, and NAC+HS groups were measured at the indicated time points post HS. Data are mean ± SD from six separate experiments per time point. **p<0.01 compared with the control group, #*p*<0.05, ##*p*<0.01 compared with the HS group. NAC, N-acetylcysteine; vWF, von Willebrand factor; EGCX, endothelial glycocalyx; HUVECs, human umbilical vein endothelial cells; W-P bodies, Weibel-Palade bodies.

We utilized a heat-stressed HUVEC model to further examine whether HS initiates EGCX damage, thereby causing vWF release; more importantly, whether application of NAC is capable of ameliorating EGCX injury and preventing vWF release from the heat-stressed HUVECs. At 2 h of HS, the shedding and degradation of EGCX in HUVECs were detected by TEM, which became more obvious at 6 h of HS and reached the maximal levels at 24 h of HS (**Figure 8A**). Immunofluorescent staining showed that the vWF molecule represented by the green fluorescence was exclusively distributed in spots i.e., the Weibel-Palade (W-P) bodies in the cytoplasm of HUVECs at 0 h of HS or in the control group, whereas ribbons or strips of vWF, representing the release of vWF, were formed on the surface of or within HUVECs after subjected to HS (**Figure 8B**). Furthermore, vWF levels in the cell culture supernatants increased substantially upon HS and reached its peak level at 24 h of HS (*p*<0.01 versus control group) (**Figure 8C**). Critically, application of NAC efficiently alleviated heat stress-induced EGCX injury in HUVECs (**Figure 8A**) and subsequently, strongly prevented vWF release from the heat-stressed HUVECs (**Figures 8B, C**), suggesting that NAC attenuates the release of vWF from the heat-stressed vascular endothelial cells *via* the amelioration of EGCX injury, thereby mitigating both the inflammatory response and coagulation.

### NAC protects against EGCX damage by attenuating ROS generation in heat-stressed or H_2_O_2_-stimulated endothelial cells

**Figure 9:**
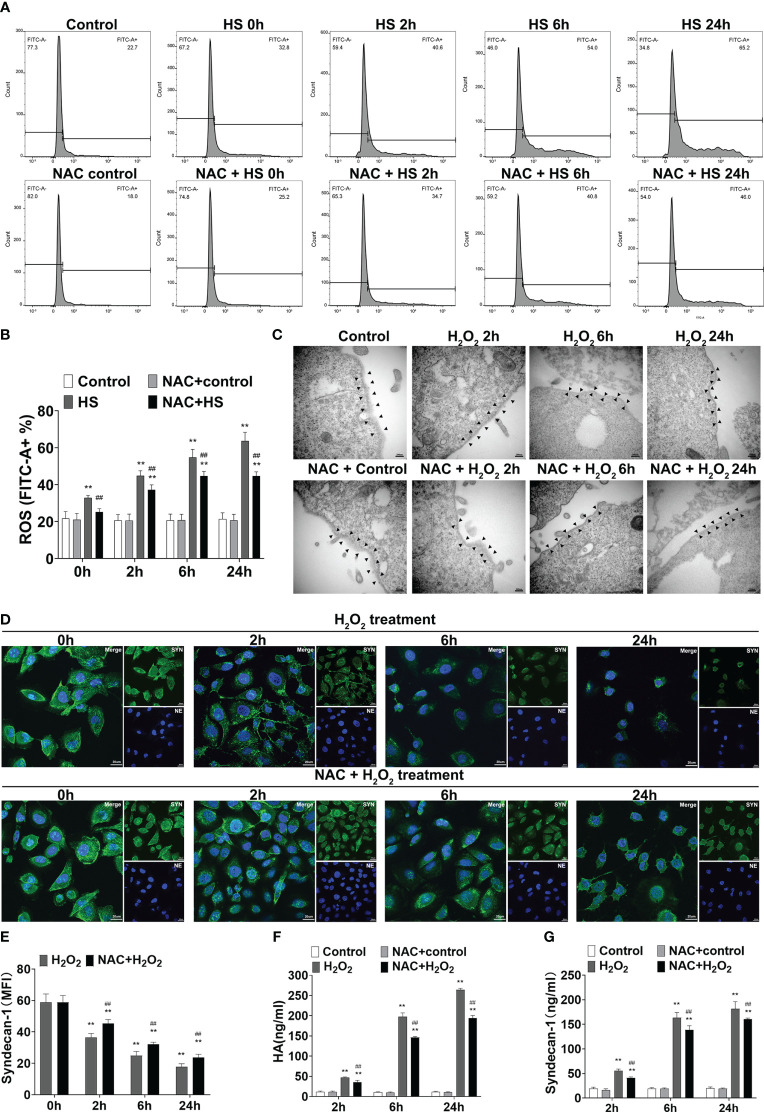
NAC attenuates ROS production in the heat-stressed vascular endothelial cells and protects against EGCX damage in H_2_O_2_-treated HUVECs. HUVECs pretreated with or without NAC were subjected to HS for 0, 2, 6, and 24 h, and ROS production in the different groups was detected by FACScan analysis **(A, B)**. HUVECs pretreated with or without NAC were incubated with H_2_O_2_ (100 μM) for 0, 2, 6, and 24 h, and cells collected at the indicated time points were assessed for EGCX under transmission electron microscope **(C)** and syndecan-1 expression by immunofluorescent staining with anti-syndecan-1 Ab and Alexa Flour 488-conjugated secondary Ab **(D, E)**. Cell nuclei were stained with Hoche 33258. Bars represent 200 nm in **(C)** and 25 µm in **(D)**. HA **(F)** and syndecan-1 **(G)** levels in the culture supernatants collected from the different groups were measured at the indicated time points after HUVECs were incubated with H_2_O_2_. Data are mean ± SD from six separate experiments per time point. **p<0.01 compared with the control group, ##*p*<0.01 compared with the HS group or H_2_O_2_ group. NAC, N-acetylcysteine; EGCX, endothelial glycocalyx; HUVECs, human umbilical vein endothelial cells; H_2_O_2_, hydrogen peroxide; ROS, reactive oxygen species; HA, hyaluronic acid.

To clarify the possible mechanism(s) by which NAC alleviates vascular EGCX injury in HS rats, we first examined whether NAC is capable of attenuating ROS generation in heat-stressed vascular endothelial cells. FACScan analysis showed that ROS levels in HUVECs increased significantly upon heat stress in a time-dependent manner (*p*<0.01 versus control group), whereas pretreatment with NAC substantially attenuated ROS generation in heat-stressed HUVECs (*p*<0.01 versus HS group) (**Figures 9A, B**). We further asked whether stimulation of HUVECs with H_2_O_2_, an important component of ROS, could directly cause EGCX damage and moreover, whether application of NAC alleviates H_2_O_2_-induced EGCX injury. As revealed by TEM, the degree of EGCX damage manifested by the destructed integrity of EGCX with gradually decreased thickness and even disappearance of EGCX, increased substantially and continuously following the extension of the stimulation time of HUVECs with H_2_O_2_ (**Figure 9C**). Immunofluorescent staining showed that syndecan-1 expression represented by the green fluorescence in the H_2_O_2_-stimulated HUVECs was gradually decreased, and the lowest syndecan-1 expression was evident at 24 h post H_2_O_2_ stimulation (**Figure 9D, E**). Two EGCX biomarkers, syndecan-1 and HA levels in the cell culture supernatants also increased markedly in H_2_O_2_-stimulated HUVECs and reached their highest levels at 24 h post H_2_O_2_ stimulation (**Figures 9F, G**). Importantly, pretreatment with NAC strongly protected against H_2_O_2_-initiated EGCX damage (**Figure 9C**), prevented H_2_O_2_-induced loss of syndecan-1 (**Figures 9D, E**), and attenuated the release of both syndecan-1 and HA from H_2_O_2_-stimulated HUVECs (**Figures 9F, G**).

## Discussion

In this study, analysis of the clinical data demonstrated that the presence of DIC was closely related to the higher risk of death in HS patients. To further explore the underlying mechanism(s) by which DIC occurs during the process of HS, we examined the characteristics of HS-initiated coagulation disorders in a rat HS model. Critically, rats subjected to heat stress experienced a transition from hypercoagulability to hypocoagulability, a typical process of DIC, during the development of HS. Notably, pulmonary capillary EGCX was severely damaged in HS rats, and circulating syndecan-1 and HA, two markers of EGCX, correlated positively with increased serum levels of endothelial injury marker endothelin-1, coagulation markers, and proinflammatory cytokines, indicating that EGCX injury is not only responsible for causing endothelial cell damage but also predominantly involved in the development of coagulation disorders. Importantly, pretreatment with NAC effectively alleviated EGCX damage with attenuated proinflammatory cytokine release and improved hypo-coagulable state in HS rats. Mechanically, HS initiated ROS generation in vascular endothelial cells, while ROS could directly cause EGCX damage. Remarkably, NAC protected against EGCX injury by attenuating ROS production in heat-stressed or H_2_O_2_-stimulated HUVECs. These results indicate that protection against EGCX injury might be an important target for preventing HS-associated coagulopathy, thereby improving the prognosis of HS.

The clinical diagnosis of HS after heat exposure is generally based on a marked increase in core body temperature in combination with the presence of central nervous system abnormalities, i.e., the Bouchama criteria (1). Since it is difficult to judge the central nervous system function in animals, the determination of the occurrence of HS in animals is often only according to an elevated core body temperature (30). However, our previous work found that simply using an increase in the core body temperature as the marker for the onset of HS in a rat HS model, there were substantial differences in the development and progression of coagulation disorders among HS rats, due to their different capability of tolerance to heat stress; therefore, we established a non-anesthetized rat HS model where we selected the time point at which SBP starts to decline as an indicator for the occurrence of HS (31). The advantage of this rat model is that the modeling conditions and standards conform to the pathogenesis of clinical severe HS, and moreover, HS rats established by this method uniformly display comparable coagulation disorders observed in our preliminary experiments (data not shown). In the present study, we used this model to track the kinetic changes of coagulation parameters before and after the onset of HS. We found that at the Tc of 43°C, just before the onset of HS, the only index indicating a hypercoagulability tendency was the significantly increased CR, suggesting that CR may serve as a sensitive marker for detecting the hypercoagulable state at the early stage of HS. On the other hand, APTT started to prolong before the onset of HS and reached its maximum level at 24 h of HS, indicating that APTT is more sensitive for monitoring a growing trend of the consumption in coagulation factors and hypocoagulability caused by DIC in HS, which is consistent with the previously reported (6). Notably, PF exhibited progressive dysfunction from before the onset of HS until after the onset of HS and reached its lowest limit at 24 h of HS; meanwhile, plasma vWF began to increase from the onset of HS and reached the highest level at 24 h of HS. Having considered a recent new view that platelet-vWF interaction is the primary pathway responsible for the formation of microthrombi during septic coagulopathy (32–34), we speculate that heat stress-initiated endothelial cell damage causes exocytosis of vWF and the released vWF stimulates platelet aggregation to form the microthrombi, thereby resulting in platelet dysfunction. Collectively, the present study replicated a rat HS model complicated with abnormal coagulation, characterized by a rapid transition from hypercoagulability and platelet activation before the onset of HS to hypocoagulability and platelet dysfunction after the onset of HS. Thus, this rat HS model used in the present study displays the staged progression of HS-induced coagulopathy more completely than other animal HS model used by the previous studies.

Currently, the precise mechanism(s) by which HS leads to DIC is unclear, and endothelial damage may play an important role in HS-induced DIC (1, 35). In a baboon HS model, it has been confirmed that inflammation, microvascular damage, endothelial cell apoptosis, and tissue factor release are all closely related to the occurrence of DIC (36). EGCX is a gel structure covering the surface of endothelial cells with a variety of protective functions, and under stress conditions such as inflammation and ischemia-reperfusion it can be degraded or shed to expose endothelial adhesion molecules and to release vWF, vWF, synthesized in the W-P bodies of vascular endothelium, is a multifunctional acute glycoprotein, and one of its main functions is to promote the adhesion and aggregation of platelets and neutrophils, thus initiating the development of micro-thrombosis (37–41). In this study, we found that plasma vWF in HS rats elevated sharply after the start of HS with a peak level at 24 h of HS, consistent with the occurrence of EGCX injury. Moreover, plasma vWF levels after HS correlated positively with standard and viscoelastic coagulation indexes, biomarkers for coagulation function and EGCX damage, as well as proinflammatory cytokines (data not shown), suggesting a strong correlation of circulating vWF with coagulation and inflammation during the process of HS. To this end, we further examined whether HS initiates EGCX damage, thereby causing vWF release using a heat-stressed HUVEC model and found that HS induced EGCX injury and resulted in massive release of vWF from the heat-stressed vascular endothelial cells. Both syndecan-1 and HA are the main components derived from the shedding of the damaged EGCX and play procoagulant and proinflammatory effects after entering the circulation (42, 43). In an LPS-induced rat sepsis model, levels of degraded components from EGCX correlated closely with plasma TNF-α, IL-6, and coagulation biomarkers, whereas administration of unfractionated heparin (UFH) substantially alleviated EGCX injury and coagulopathy (44). By replicating an anesthetized rat HS model, Kensuke et al. demonstrated that EGCX injury, as manifested by a significant increase in plasma syndecan-1 levels, was associated with the mortality rate at 2 h after resuscitation (45). In the present study, we also found that changes of plasma syndecan-1 and HA in HS rats were parallel to the time of coagulation and platelet activation and correlated positively with the levels of coagulation markers, suggesting that EGCX injury may be involved in HS-initiated coagulation disorders and early protection of EGCX is therefore critical for improving prognosis of HS.

A large number of studies have shown that heat stress results in substantial release of inflammatory mediators and a significant increase in ROS generation, which are important factors in causing damage to EGCX (46–48). As both an anti-inflammatory and an antioxidant agent, NAC has the effect of inhibiting the release of proinflammatory cytokines such as TNF-α and IL-8, while supplementing exhausted glutathione to exert the free radical scavenging effect, making it as a potential drug of choice for sepsis and acetaminophen-induced poisoning (49–52). The present study aimed to investigate the possible mechanism(s) by which NAC protects against vascular EGCX injury in HS rats. We found that heat stress resulted in time-dependent increases in ROS generation and NAC efficiently attenuated ROS production in heat-stressed HUVECs. Critically, stimulation of HUVECs with H_2_O_2_, an important component of ROS, directly caused EGCX damage as confirmed by TEM and immunofluorescent staining. Remarkably, NAC strongly alleviated H_2_O_2_-induced EGCX damage with restored syndecan-1 expression and attenuated release of syndecan-1 and HA, indicating that NAC-afforded protection against HS-induced EGCX damage is, at least in part, *via* its ROS scavenging effect.

Our study has the following limitations which are desirable for further clarification. First, this study only analyzed the changes of coagulation indexes for surviving rats at various time points after HS and did not compare the differences of DIC parameters between surviving and dead rats. Second, only the protection afforded by preventive intervention of NAC during HS was explored in the present study, and the therapeutic effect of NAC on HS rats certainly needs to be further investigated. Third, more efforts should be made to examine the effect of NAC on organ functions closely related to coagulation disorder and survival rate. In short, our results demonstrate that NAC exerts its ability in controlling HS-initiated progression of coagulation disorders. Nevertheless, further work is required to determine whether NAC-afforded protection against EGCX injury is through its direct effect or indirect effect of its anti-inflammatory and antioxidant capabilities.

## Data availability statement

The original contributions presented in the study are included in the article/supplementary material. Further inquiries can be directed to the corresponding authors.

## Ethics statement

The studies involving human participants were reviewed and approved by the ethics committee of general hospital of southern theatre command and informed consent was waived because it was a retrospective observational study. Written informed consent for participation was not required for this study in accordance with the national legislation and the institutional requirements. The animal study was reviewed and approved by the institutional animal care and use committee of general hospital of southern theatre command and complied with the animal welfare act.

## Author contributions

NP, LS, HL, JW, and JL conceived and designed the experiments. YG, FY, WC, BY, and SL performed the experiments. JO, YW, YT, JL and HL participated in the statistical analysis. NP, JW, and JL wrote the manuscript. All authors contributed to the article and approved the submitted version.
